# MRI Evaluation of Fetoscopic Endoluminal Tracheal Occlusion for an Isolated Left Congenital Diaphragmatic Hernia and Clinical Outcomes of Neonates after Delivery: Five Case Reports and Literature Review

**DOI:** 10.2174/0115734056402208250923101426

**Published:** 2025-10-02

**Authors:** Wei Tang, Yan Zhou, Wei Tian, Chuanfei Xie, Xiaojie Lan, Jiayan Ming, Song Peng

**Affiliations:** 1 Chongqing Health Center for Women and Children, Women and Children’s Hospital of Chongqing Medical University, Chongqing 401147, China; 2 NHC Key Laboratory of Birth Defects and Reproductive Health, Chongqing 401147, China; 3 Chongqing Municipal Health Commission Key Laboratory of Perinatal Medicine, Chongqing 401147, China

**Keywords:** Fetoscopic Endoluminal Tracheal Occlusion (FETO), Congenital diaphragmatic hernia, Magnetic Resonance Imaging (MRI), Lung volume, Maternal body habitus, Fetal MRI

## Abstract

**Introduction:**

Prenatal intervention with fetoscopic endoluminal tracheal occlusion (FETO) using a balloon can stimulate lung growth and improve neonatal survival for moderate and severe congenital diaphragmatic hernia (CDH). Quantitative parameters measured on magnetic resonance imaging (MRI) can guide the treatment of CDH and evaluate changes after FETO treatment.

**Case presentation:**

We reported on five cases of isolated left congenital diaphragmatic hernia (CDH) in fetuses who underwent FETO surgery. We conducted a comparison of the MRI images before and after FETO treatment and analyzed the correlation between the observed changes and the clinical outcomes of the neonates after delivery.

**Conclusion:**

MRI can precisely provide the anatomical details of CDH and quantitatively analyze changes in fetal lung volume before and after FETO surgery.

## 1 INTRODUCTION

Congenital diaphragmatic hernia (CDH) is a congenital defect in which the diaphragm does not fully close, allowing abdominal organs to herniate into the fetal chest. This pathological displacement results in compression of the developing lungs and heart, leading to pulmonary hypoplasia and impaired pulmonary vascular development [1]. Consequently, neonates with CDH, particularly those with severe forms, face life-threatening respiratory insufficiency and persistent pulmonary hypertension of the newborn [2]. Prenatal intervention via fetoscopic endoluminal tracheal occlusion (FETO) has emerged as a therapeutic strategy to mitigate these sequelae. By inducing tracheal occlusion with a balloon device, FETO promotes fetal lung growth through the retention of pulmonary fluid, thereby enhancing the prospects for neonatal survival [3].

Current diagnostic imaging modalities for CDH primarily rely on ultrasonography (US) and fetal magnetic resonance imaging (MRI) [4-7]. Fetal MRI is widely recognized as superior to both 2D and 3D US in diagnosis, pre-operative planning, and follow-up [8, 9]. This advantage stems from its inherent technical strengths, including superior soft tissue contrast, a large field of view unimpeded by fetal position or maternal body habitus, and relative insensitivity to acoustic shadowing from bone or bowel gas [10]. Multiple quantitative imaging features (*e.g.*, observed-to-expected FLV [o/e FLV], percent predicted lung volume (PPLV), and liver herniation quantification) can be derived from MRI examinations [11, 12]. These imaging features are instrumental in guiding prenatal management decisions, including FETO candidacy, and in evaluating the morphological response of the fetal lungs and thoracic cavity following FETO intervention [13].

The utility of MRI in the initial evaluation and preoperative planning of CDH is well-documented in numerous studies [11-14]. However, there is limited MRI data on the longitudinal changes following FETO balloon placement and their connection to postnatal clinical outcomes. In our study, we collected and analyzed longitudinal fetal MRI data from a cohort of five fetuses with CDH who underwent FETO in our hospital between November 2023 and August 2023. We conducted a detailed comparative analysis of key MRI parameters before and after the FETO intervention and investigated potential correlations between the observed MRI changes and the early postnatal clinical outcomes of the neonates.

## 2 METHODS

Fetal MRI was performed using a 1.5-T whole-body clinical scanner (Ingenia; Philips Healthcare, The Netherlands). The T2-weighted images were transmitted to a picture archiving and communication system (PACS) (Lanwon Medical Systems, China), and processed using the configured image processing and measurement tools in the PACS system to calculate the following parameters: total fetal lung volume (TFLV), observed-to-expected TFLV (o/e TFLV), percent predicted lung volume (PPLV), observed-to-expected lung-to-head ratio (o/e LHR), lung-to-liver signal intensity ratio (LLSIR), and the percentage of liver herniation into the thoracic cavity (%LH). These parameter calculations were completed by a prenatal MRI diagnostic physician with 5 years of experience, utilizing methods documented in the literature [12, 13].

## 3 CASE PRESENTATION

All procedures performed in this study were in accordance with the ethical standards of the institutional committee (approval No. 2023 No. 062) and the principles outlined in the Declaration of Helsinki. Written informed consent was obtained from the patients. The clinical characteristics and MRI quantitative indicators of the five cases with CDH are shown in Tables **1** and **2**.

### 3.1 Case 1

**Fig. (1):**
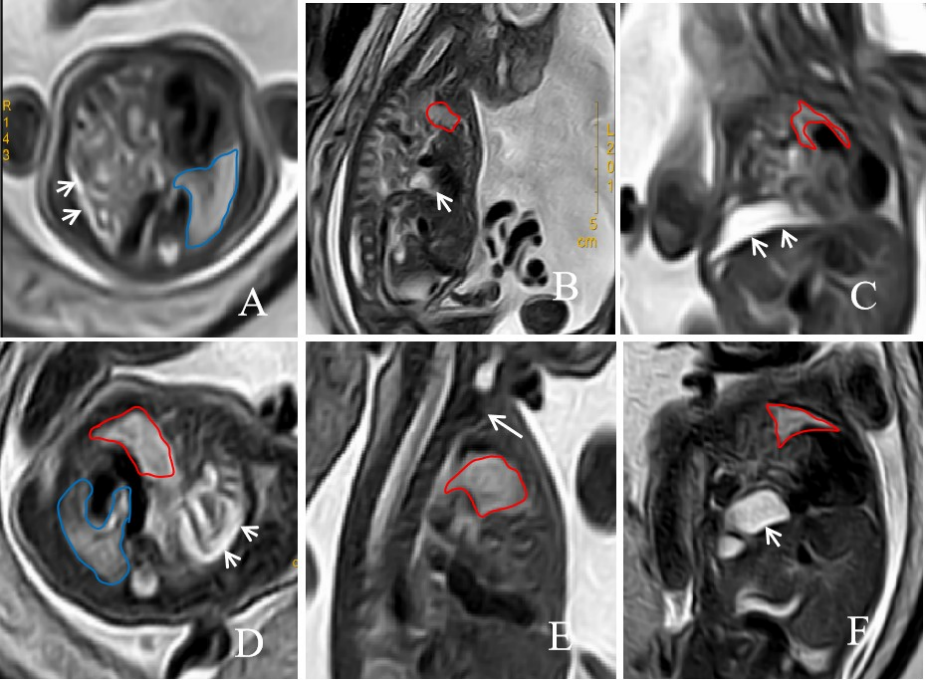
A 43-year-old pregnant woman, the fetus with left CHD on prenatal MRI. The axial, sagittal, and coronal T2-weighted images at 28w5d of gestation(**A**, **B**, and **C**) before FETO treatment, and at 33w0d of gestation after FETO treatment(**D**, **E**, and **F**) showed abdominal organs, such as the stomach and intestines, herniated into the chest cavity(short arrow). A T2-weighted image on a sagittal plane showed that (E, long arrow). The area delineated by the red line represents the left lung that has herniated into the chest cavity, while the area outlined by the blue line is the normal right lung.

A 43-year-old East Asian multipara was referred for confirmation of a left congenital diaphragmatic hernia (CDH) in the fetus (Fig. **1**). Prenatal MRI at 28w5d of gestation revealed a severe left CDH with the stomach, intestinal canal, spleen, and part of the left kidney herniating into the thorax through the diaphragmatic defect, with the following parameters: TFLV 9.64 ml, o/e TFLV 14.15%, PPLV 11.48%, o/e LHR 10.94%, and LLSIR 1.51. After multidisciplinary counseling, the parents opted for fetoscopic endoluminal tracheal occlusion (FETO), and preoperative amniocentesis showed a normal 46,XY karyotype. At 29w0d, a fetal endoscope was inserted through the maternal abdominal wall and uterus under ultrasound guidance, and a balloon was placed in the mid-lower trachea approximately 1–2 cm above the carina to achieve temporary occlusion. A follow-up MRI at 33 weeks 0 days demonstrated correct balloon placement and improved left lung development, with parameters of TFLV 16.82 ml, o/e TFLV 24.79%, PPLV 19.23%, o/e LHR 20.02%, and LLSIR 1.60.

At 33 weeks and 2 days of gestation, premature preterm rupture of membranes (PPROM) occurred. It was recommended to perform fetoscopic removal of the balloon immediately and to begin fetal protection treatment to prolong the pregnancy as much as possible. Under the guidance of a fetal endoscope, a bronchoscope was inserted transorally and advanced to the tracheal balloon. A thin needle was then used to puncture the balloon, enabling gentle removal of the deflated balloon and catheter from the trachea. At 34 weeks of gestation, the pregnant woman experienced lower abdominal distension and pain. Fetal heart rate monitoring showed frequent fluctuations. The attending physician decided to perform a cesarean section (C-section), successfully delivering a male newborn who weighed 1680 grams. After birth, the newborn had irregular breathing and cyanotic skin color. At 1, 5, and 10 minutes, the Apgar scores were 8, 8, and 10, respectively. The newborn received treatments including mechanical ventilation, pulmonary artery decompression, volume expansion, anti-infection therapy, intravenous nutrition, and fresh frozen plasma infusion. On the second day after birth, the newborn's condition gradually worsened. Despite rescue efforts, the newborn did not survive due to severe CDH and pulmonary hemorrhage.

### 3.2 Case 2

**Fig. (2):**
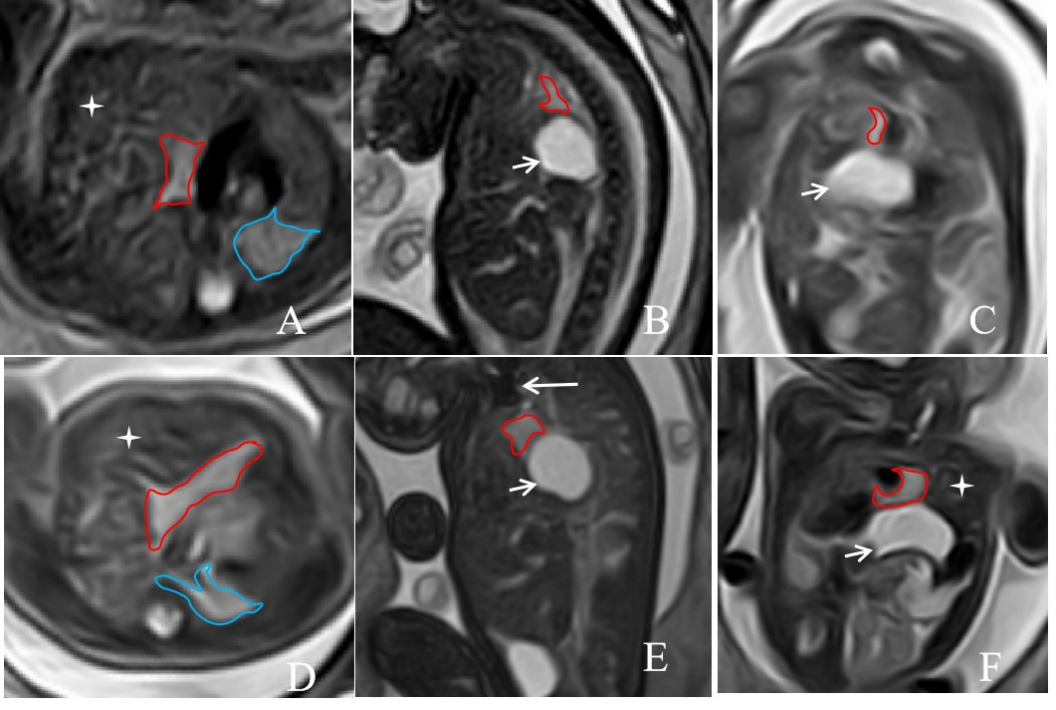
A 38-year-old pregnant woman, the fetus with left CHD on prenatal MRI. The axial, sagittal, and coronal T2-weighted images at 28w0d of gestation(**A**, **B** and **C**) before FETO treatment, and at 33w5d of gestation after FETO treatment(**D**, **E** and **F**) showed abdominal organs, such as the stomach, intestines, and partial left lobe of liver, herniated into the chest cavity(short arrow and cross Star). A T2-weighted image on a sagittal plane showed that a low-signal balloon was inserted into the trachea above the carina during FETO surgery (E, long arrow). The area delineated by the red line represents the left lung that has herniated into the chest cavity, while the area outlined by the blue line is the normal right lung.

A 38-year-old East Asian multipara was referred for confirmation of a left CDH in a fetus. A prenatal MRI of the fetus performed at 28w0d of gestation showed a severe left CDH with the stomach, intestinal canal, spleen, and the partial left lobe of the liver herniating into the thorax through the left diaphragmatic defect (Fig. **2A**–**F**). The indicators measured on MRI were as follows: TFLV: 10.03 ml; o/e TFLV: 17.97%; PPLV: 12.62%; o/e LHR: 15.01%; LLSIR: 2.03; Proportion of liver herniation into the chest cavity (%LH): 28.83%. The fetus underwent a successful FETO procedure at 29w5d under ultrasound guidance. Preoperative amniocentesis revealed a normal 46,XY karyotype. A follow-up MRI performed at 33w5d of gestation demonstrated correct balloon placement (Fig. **2E**) and improved development of the left lung, with TFLV: 19.54 ml; o/e TFLV: 25.82%; PPLV: 20.64%; o/e LHR: 22.19%; LLSIR: 2.36; Proportion of liver herniation into the chest cavity (%LH): 23.56%.

At 34 weeks and 6 days of pregnancy, the pregnant woman experienced premature rupture of membranes (PPROM) and was advised to have a cesarean section. After the delivery of the fetal head, the fetus underwent fiberoptic bronchoscopy and fetal endoscopy to remove the balloon through the mouth without cutting the umbilical cord. Once the balloon was removed, tracheal intubation and positive pressure airway management were performed, with an FIO2 of 100%. Subsequently, the umbilical cord was cut, and a female newborn was delivered intact with a weight of 2200 g. After birth, the newborn exhibited cyanosis throughout the body, had no spontaneous breathing, no detectable pulse, and no audible heart sounds. At 1, 5, and 10 minutes, the Apgar scores were 1, 1, and 2, respectively. Continuous positive airway pressure ventilation (FIO2 100%) and extracardiac compression were performed immediately. Emergency bedside cranial US indicated cerebral hemorrhage, and emergency blood gas analysis suggested type 2 respiratory failure. Despite rescue measures including expansion, acid-base correction, and blood pressure support, the newborn ultimately died within 3 hours after birth due to severe CDH and respiratory and circulatory failure.

### 3.3 Case 3

**Fig. (3):**
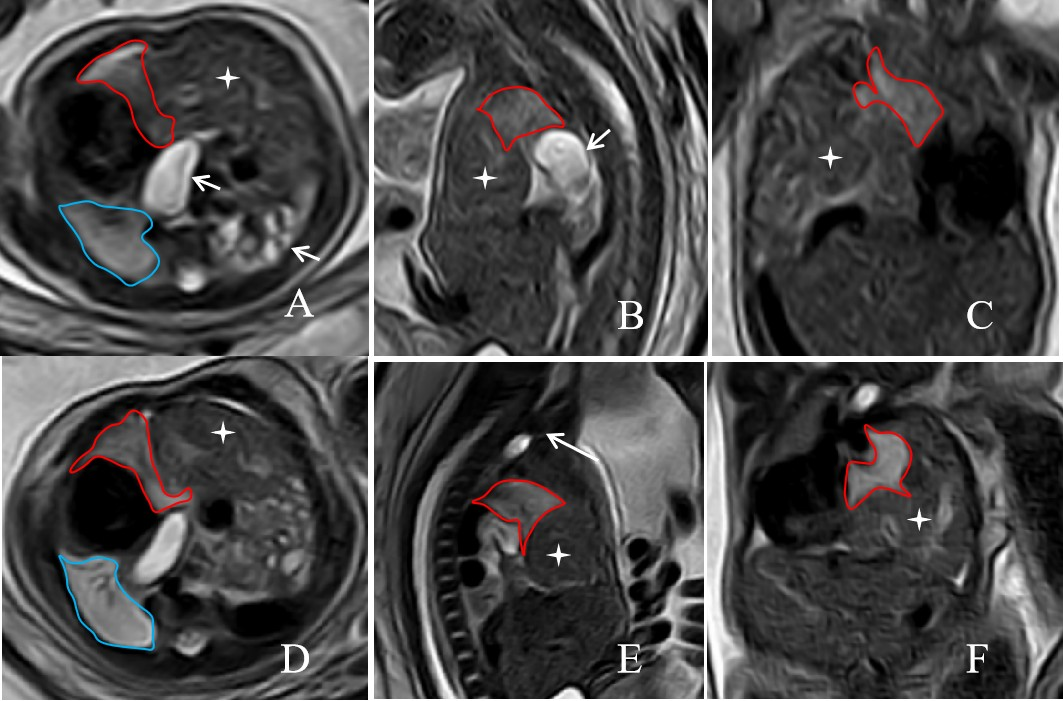
A 33-years-old pregnant woman, the fetus with left CHD on prenatal MRI. The axial, sagittal, and coronal T2-weighted images at 29w3d of gestation(**A**, **B** and **C**) before FETO treatment, and at 34w3d of gestation after FETO treatment(**D**, **E** and **F**) showed abdominal organs, such as the stomach, intestines, and partial left lobe of liver, herniated into the chest cavity(short arrow and cross Star). A T2-weighted image on a sagittal plane showed that a low-signal balloon was inserted into the trachea above the carina during FETO surgery (E, long arrow). The area delineated by the red line represents the left lung that has herniated into the chest cavity; the area outlined by the blue line is the normal right lung.

A 33-year-old East Asian multipara was referred for confirmation of a left CDH in a fetus. A prenatal MRI of the fetus performed at 29w3d of gestation showed a severe left CDH with the stomach, intestinal canal, and the partial left lobe of the liver herniating into the thorax through the left diaphragmatic defect (Fig. **3A**–**F**). The indicators measured on MRI were as follows: TFLV: 11.1 ml; o/e TFLV: 20.12%; PPLV: 15.54%; o/e LHR: 16.05%; LLSIR: 1.96; Partial left lobe of liver herniation into the chest cavity (%LH): 29.06%. The fetus underwent a successful FETO procedure at 29w6d under ultrasound guidance. Preoperative amniocentesis revealed a normal 46,XY karyotype. Dexamethasone was administered for one course after FETO surgery to promote fetal lung maturation. A follow-up MRI performed at 34w3d demonstrated correct balloon placement and improved development of the left lung (Fig. **3E**), with TFLV: 24.3 ml; o/e TFLV: 27.03%; PPLV: 19.87%; o/e LHR: 24.3%; LLSIR: 2.25; Proportion of liver herniation into the chest cavity (%LH): 24.2%.

Fetoscopic removal of the balloon was performed at 35 weeks of gestation. A male newborn weighing 2980 g was delivered by C-section at 37 weeks and 4 days of gestation without premature rupture of membranes (PPROM), with Apgar scores of 8, 8, and 8 points at 1, 5, and 10 minutes. The newborn presented with tachypnea and a distinctive three-concave sign. At 9 hours after birth, the newborn's oxygen index (OI) value reached 58. After evaluation by the extracorporeal membrane oxygenation (ECMO) team, ECMO treatment was planned. The newborn's condition improved after 2 days of ECMO supportive treatment, with a stable internal environment, stable airflow, and balanced inflow and outflow. The newborn underwent diaphragmatic hernia repair with ECMO support three days after birth. During hospitalization, additional symptomatic supportive treatments were provided, such as pulmonary artery pressure reduction, infection control, fluid expansion, and correction of electrolyte imbalances. Unfortunately, the newborn ultimately died 14 days after birth because of severe CDH and respiratory and circulatory failure.

### 3.4 Case 4

**Fig. (4):**
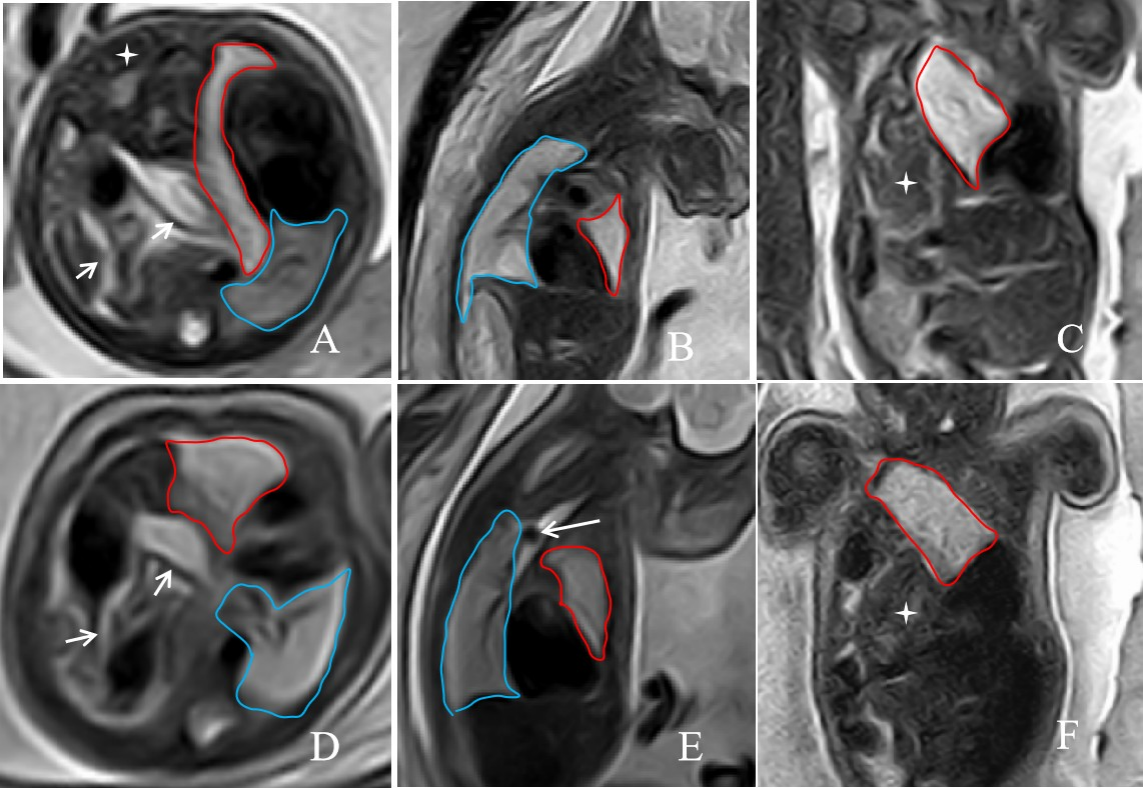
A 28-years-old pregnant woman, the fetus with left CHD combined with congenital pulmonary cystadenoma on prenatal MRI. The axial, sagittal, and coronal T2-weighted images at 28w1d of gestation(**A**, **B** and **C**) before FETO treatment, and at 35w of gestation after FETO treatment(**D**, **E** and **F**) showed abdominal organs, such as the stomach, intestines, and partial left lobe of liver, herniated into the chest cavity (short arrow and cross Star). A sagittal T2-weighted image (**E**) showed that a low-signal balloon was inserted into the trachea above the carina during FETO surgery(E, long arrow). The area delineated by the red line represents the congenital cystadenoma occurring in the left lung, while the area outlined by the blue line represents the normal right lung.

A 28 years-old east Asian multipara was referred for confirmation of left CDH of a fetus combined with congenital pulmonary cystadenomatoid malformation. A prenatal MRI of the fetus performed at 28w1d of gestation showed a severe left CDH with stomach, intestinal canal, part of the spleen, and left lobe of liver herniating into the thorax through the left diaphragmatic defect site (Fig. **4A**-**F**). In addition, an irregular mass was observed in the left chest cavity on prenatal MRI, showing lower signal intensity on T1-weighted images and higher signal intensity on T2-weighted images compared to the normal lung tissue on the opposite side, with a size of 36.1 × 22.3 × 36.5 mm (Fig. **4** , the area delineated by the red line). Based on the MRI signal characteristics, the mass was diagnosed as congenital pulmonary cystadenomatoid malformation. The presence of congenital pulmonary cystadenomatoid malformation complicates the assessment of the left chest cavity on MRI, making it difficult to ascertain whether any normal lung tissue remains. However, an ultrasound examination suggested that only a minimal amount of healthy lung tissue exists in the left chest cavity. For this patient, measuring the left lung volume on MRI included the volume of the congenital pulmonary cystadenomatoid malformation. The indicators measured on MRI were as follows: TFLV: 20.9 ml; o/eTFLV: 23.7%; PPLV: 20.94%; o/eLHR: 24.49%; LLSIR: 2.41; partial left lobe of liver herniation into the chest cavity (%LH): 26.5%. The pregnant woman underwent a successful FETO procedure at 28w4d under ultrasound guidance. Amniocentesis was performed preoperatively and resulted in a normal 46,XY karyotype. A second follow-up MRI examination performed at 35w0d demonstrated correct placement of the balloon and improved development of the left lung (Fig. **4E**), with a TFLV: 29.41 ml; o/eTFLV: 28.8%; PPLV: 29.10%; o/eLHR: 28.31%; LLSIR: 3.7; proportion of liver herniation into the chest cavity (%LH): 15.88%.

Fetoscopic removal of the balloon was performed at 35 weeks and 2 days of gestation. At 35 weeks and 4 days of pregnancy, the pregnant woman experienced premature rupture of membranes (pPROM) and was advised to have a cesarean section. A female newborn weighing 2400 g was delivered by C-section at 35 weeks and 4 days of gestation, with Apgar scores of 6, 5, and 10 points at 1, 5, and 10 minutes. This newborn underwent endotracheal intubation immediately and was connected to a ventilator for assisted ventilation after birth because of shortness of breath.

At two days after birth, a transabdominal diaphragmatic hernia repair was performed under general anesthesia, along with closed thoracic drainage. The newborn continued to receive ventilator-assisted ventilation for 17 days and non-invasive ventilation for 5 days after surgery, and received supportive treatments such as blood transfusion to correct anemia, albumin supplementation, and intravenous nutrition. After 34 days of hospitalization, the patient was cured and discharged.

### 3.5 Case 5

**Fig. (5):**
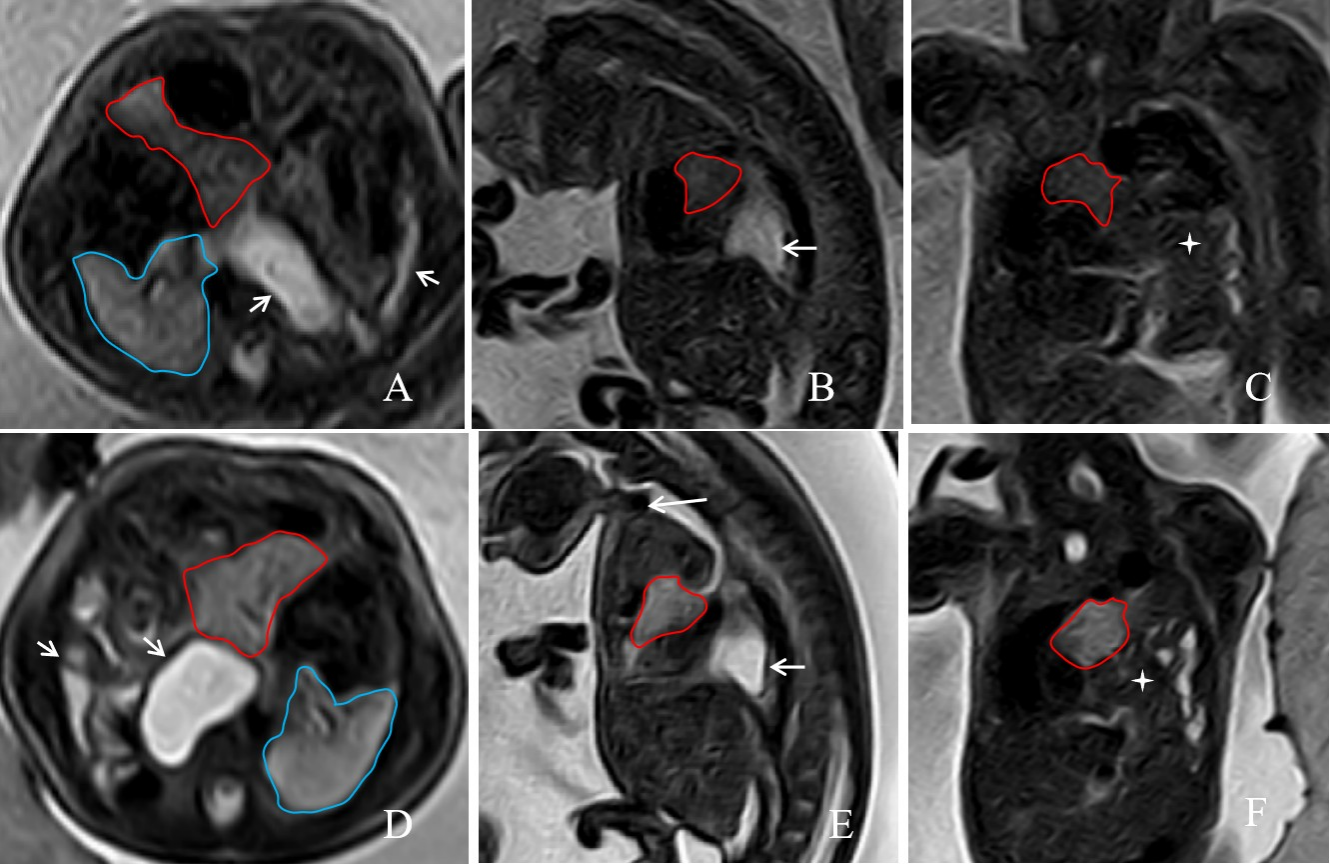
A 32-year-old pregnant woman, the fetus with left CHD on prenatal MRI. The axial, sagittal, and coronal T2-weighted images at 29w5d of gestation(**A**, **B** and **C**) before FETO treatment, and at 32w of gestation after FETO treatment(**D**, **E** and **F**) showed abdominal organs, such as the stomach, intestines, and partial left lobe of liver, herniated into the chest cavity(short arrow and cross Star). A sagittal T2-weighted image showed that a low-signal balloon was inserted into the trachea above the carina during FETO surgery(E, long arrow). The area delineated by the red line represents the left lung that has herniated into the chest cavity, while the area outlined by the blue line is the normal right lung.

A 32 years-old East Asian multipara was referred for confirmation of left CDH of a fetus. A prenatal MRI of the fetus performed at 29w5d of gestation showed a severe left CDH with stomach, intestinal canal, spleen, and partial left lobe of liver herniating into the thorax through the left diaphragmatic defect site (Fig. **5A**-**F**). The indicators measured on MRI were as follows: TFLV:12.41 ml;o/eTFLV:16.99%;PPLV:14.63%;o/eLHR:20.39%;LLSIR:1.99;Partial left lobe of liver herniation into the chest cavity (%LH):16.83%. The fetus underwent a successful FETO procedure at 29w6d under ultrasound guidance. Amniocentesis was performed preoperatively and resulted in normal 46,XY karyotype. A second follow-up MRI examination performed at 32w0d demonstrated a correct placement of the balloon and improved development of the left lung (Fig. **5E**), with a TFLV:18.85 ml;o/eTFLV:26.32%;PPLV:20.47%;o/eLHR: 31.86%;LLSIR:2.62;Proportion of liver herniation into the chest cavity (%LH):14.76%.

At 32 weeks and 2 days of pregnancy, the pregnant woman experienced premature rupture of membranes (pPROM). It was recommended to begin fetal protection treatment to prolong the pregnancy for as long as possible, facilitating the development and maturation of the fetal lungs. Fetoscopic removal of the balloon was performed at 34 weeks of gestation. A male newborn weighing 2200 g was delivered by C-section at 35 weeks of gestation, with Apgar scores of 6, 7, and 8 points at 1, 5, and 10 minutes, presenting with tachypnea. The newborn underwent a transabdominal diaphragmatic hernia repair and closed thoracic drainage under general anesthesia one day after birth. During hospitalization, various supportive treatments were implemented, including mechanical ventilation, pulmonary artery pressure reduction, infection control, fluid expansion, and correction of electrolyte imbalances. Unfortunately, the newborn ultimately died two days after birth due to severe CDH and respiratory and circulatory failure.

## 4 DISCUSSION

The criteria for classifying CDH severity can rely on one indicator or a combination of two indicators. Style *et al.* [15] reported that the combination of the o/e TFLV with a cut-off value of 32% and the percentage of LH with a cut-off value of 21% can be used as criteria for classifying the severity of CDH. Similarly, Deprest *et al.* [16] noted that the combination of the o/e LHR with cut-off values of >45% (mild), 25–45% (moderate), and <25% (severe) and the presence or absence of liver herniation into the chest cavity serves as another set of criteria for determining the severity of CDH. Based on the two criteria mentioned above, the five patients fall into the category of moderate to severe CDH. As a result, these patients with CDH have been identified as candidates for FETO surgery.

In 2004, Deprest, Gratacós, and Nicolaides made significant advancements in the field by implementing FETO through the introduction of an inflatable balloon that effectively occludes the trachea [17]. The balloon is removed a few weeks prior to delivery. Tracheal occlusion leads to the accumulation of lung fluid, which causes the lungs to stretch. This stretching is followed by an increase in lung cell proliferation and a decrease in the severity of lung hypoplasia [18]. Therefore, when comparing MRI images before FETO surgery and several weeks after FETO surgery, the quantitative imaging biomarkers often demonstrated significant alterations in fetuses with CDH [19, 20]. In the five cases of CDH we reported, we observed changes in the TFLV, o/e TFLV, PPLV, o/e LHR, LLSIR, and percentage of LH measured on MRI before and after FETO surgery. While there is no statistical analysis, the data trends indicate that FETO surgery can improve the developmental status of fetal lungs in fetuses with CDH to some extent (Table **2**).

Cannie *et al.* found that the o/e TFLV after FETO surgery was most accurate at predicting survival when measured about 1 month after FETO surgery [11]. In the five cases of fetuses with CDH, the interval between FETO surgery and the second MRI examination was at least 4 weeks in four patients, while one patient had an interval of less than 4 weeks. This patient exhibited signs of premature rupture of membranes, which necessitated an MRI evaluation following FETO surgery at 32 weeks of gestation, with an interval of less than 3 weeks between the MRI examination and FETO surgery.

Data reported in some literature suggests that FETO prior to 29 weeks of gestation is associated with a better lung response and an increased postnatal survival [21, 22]. Poerwosusanta *et al.* [23] concluded that CDH repair timing does not affect mortality rates, regardless of illness severity, and surgery is performed after achieving the required physiological index in a systematic review. In fact, the optimal time for FETO surgery and the duration of the occlusion period are still uncertain, because early FETO surgery may lead to preterm delivery or premature preterm rupture of membranes [24]. Premature delivery is also an independent risk factor for survival in fetuses with CDH [25]. Among the five cases of fetuses with CDH, four underwent repair surgery when their physiological indicators met the surgical conditions, while one patient did not undergo repair surgery due to continuous deterioration of physiological indicators after birth. In addition, four pregnant women experienced premature rupture of membranes leading to preterm delivery.

In the five fetuses with CDH, only one underwent FETO surgery before 29 weeks of pregnancy. The remaining four cases had FETO surgery after 29 weeks of pregnancy. Notably, the fetus that had FETO surgery before 29 weeks survived after birth, while the other four fetuses that underwent FETO surgery after 29 weeks did not survive. The prenatal MRI of the fetus that survived after birth revealed the presence of a larger-diameter congenital pulmonary cystadenoma in the ipsilateral chest cavity of the CDH. We speculate that the congenital pulmonary cystadenoma has a certain space-occupying effect, which can to some extent counteract the compression of the heart or right lung by CDH, thereby reducing the harm to cardiovascular and lung function. This may be one of the reasons why the fetus with CDH was able to survive after birth.

Currently, FETO surgery stands as the only prenatal intervention proven to significantly improve survival outcomes for newborns with severe CDH [26]. Although FETO surgery can improve lung development and volume, the mortality of newborns with severe CDH remains high [27]. This may be because pulmonary hypertension due to pulmonary hypoplasia is the main cause of death in newborns with severe CDH.

Despite reporting relatively few cases, it is worth mentioning that we reported a case of CDH combined with a concomitant congenital cystadenoma and analyzed the possible reasons for the fetus surviving after birth. In addition, to our knowledge, there are many reports on pre- and post-FETO MRI evaluation and comparative analysis of fetuses with CDH in Europe, but there are relatively few studies from China, which can enrich the clinical evidence of fetuses with CDH in East Asia to some extent.

## 5 STUDY LIMITATION

This case report has inherent limitations. Although the five CDH cases in our study demonstrated increased lung volumes on MRI following FETO surgery compared to preoperative measurements, the small sample size precludes robust statistical analysis and limits the generalizability of the findings. Future studies should prioritize collecting a larger cohort of cases to systematically investigate the correlation between pre- and post-FETO lung volume changes and their corresponding clinical outcomes.

## CONCLUSION

In conclusion, the case report demonstrates that the FETO procedure can improve the lung volume of fetuses with CDH to a certain degree. MRI is a reliable diagnostic tool for assessing fetal lung volume after FETO surgery. Prior to FETO surgery, MRI can effectively assess the degree of fetal lung development and provide relevant anatomical details regarding deformities. Following FETO surgery, MRI is capable of monitoring both the position of the tracheal balloon and the progress of lung development.

## Figures and Tables

**Table 1:** The clinical characteristics of the five cases with CDH.

**Maternal and fetal characteristics**	**Case 1**	**Case 2**	**Case 3**	**Case 4**	**Case 5**
Maternal age (years)	43	38	33	28	32
An initial MRI at gestational age (week/days)	28w5d	28w0d	29w3d	28w1d	29w5d
A FETO procedure at gestational age (week/days)	29w0d	29w5d	29w6d	28w4d	29w6d
The second MRI at gestational age (week/days)	33w0d	33w5d	34w3d	35w0d	32w0d
Dexamethasone treatment(Y/N)	Y	Y	Y	Y	Y
Liver herniation(Y/N)	N	Y	Y	Y	Y
Spleen herniation(Y/N)	Y	Y	N	Y	Y
Kidney herniation(Y/N)	Y	N	N	N	N
Stomach/intestinal canal	Y	Y	Y	Y	Y
A fetoscopic removal of the balloon at gestational age (week/days)	33w2d	34w6d	35w0d	35w2d	34w0d
pPROM at gestational age(week/days)	33w2d	34w6d	No	35w4d	32w2d
Birth at gestational age(week/days)	34w0d	34w6d	37w4d	35w4d	35w0d
Birth weight (g)	1680	2200	2980	2400	2200
CDH repair surgery(Y/N)	N	N	Y	Y	Y
ECMO(Y/N)	N	N	Y	N	N
The time of hospitalization(day/hour)	2days	3hour	14days	34days	2days
Clinical outcome	died	died	died	survived	died

**Note:**CDH,congenital diaphragmatic hernia; FETO, fetoscopic endoluminal tracheal occlusion; pPROM, premature rupture of membranes; ECMO, extracorporeal membrane oxygenation; Y, yes; N, no.

**Table 2:** Quantitative MRI indicators of CDH before and after FETO treatment.

**Patient** **MRI indicators**	**TEVL**	**o/eTFLV**	**PPLV**	**o/eLHR**	**LLSIR**	**%LH**
Before FETO						
Patient 1	9.64ml	14.15%	11.48%	10.94%	1.51	—
Patient 2	10.03ml	17.97%	12.62%	15.01%	2.03	28.83%
Patient 3	11.1ml	20.12%	15.54%	16.05%	1.96	29.06%
Patient 4	20.9ml	23.7%	20.94%	24.49%	2.41	26.5%
Patient 5	12.41ml	16.99%	14.63%	20.39%	1.99	16.83%
After FETO						
Patient 1	16.82ml	24.79%	19.23%	20.02%	1.6	—
Patient 2	19.54ml	25.82%	20.64%	22.19%	2.36	23.56%
Patient 3	24.3ml	27.03%	19.87%	24.3%	2.25	24.2%
Patient 4	29.41ml	28.8%	29.10%	28.31%	3.7	15.88%
Patient 5	18.85ml	26.32%	20.47%	31.86%	2.62	14.76%

**Note:** CDH, congenital diaphragmatic hernia; TEVL, total fetal lung volume; o/e TFLV, observed/expected total fetal lung volume; PPLV, percent predicted lung volume; o/e LHR, observed/expected lung head ratio; LLSIR,lung liver signal intensity ratio; %LH, Proportion of liver herniation into the chest cavity.

## Data Availability

The authors confirm that the data supporting the findings of this study are available within the manuscript.

## LIST OF ABBREVIATIONS

FETO: Fetoscopic Endoluminal Tracheal Occlusion
CDH: Congenital diaphragmatic hernia
MRI: Magnetic resonance imaging
PPLV: Percent predicted lung volume
PACS: Picture archiving and communication system

## STANDARDS OF REPORTING

CARE guidelines were followed.
